# The Content and Nature of Narrative Comments on Swiss Physician Rating Websites: Analysis of 849 Comments

**DOI:** 10.2196/14336

**Published:** 2019-09-30

**Authors:** Stuart McLennan

**Affiliations:** 1 Institute of History and Ethics in Medicine Technical University of Munich Munich Germany; 2 Institute for Biomedical Ethics University of Basel Basel Switzerland

**Keywords:** physician rating websites, patient satisfaction

## Abstract

**Background:**

The majority of physician rating websites (PRWs) provide users the option to leave narrative comments about their physicians. Narrative comments potentially provide richer insights into patients’ experiences and feelings that cannot be fully captured in predefined quantitative rating scales and are increasingly being examined. However, the content and nature of narrative comments on Swiss PRWs has not been examined to date.

**Objective:**

This study aimed to examine (1) the types of issues raised in narrative comments on Swiss PRWs and (2) the evaluation tendencies of the narrative comments.

**Methods:**

A random stratified sample of 966 physicians was generated from the regions of Zürich and Geneva. Every selected physician was searched for on 3 PRWs (OkDoc, DocApp, and Medicosearch) and Google, and narrative comments were collected. Narrative comments were analyzed and classified according to a theoretical categorization framework of physician-, staff-, and practice-related issues.

**Results:**

The selected physicians had a total of 849 comments. In total, 43 subcategories addressing the physician (n=21), staff (n=8), and practice (n=14) were identified. None of the PRWs’ comments covered all 43 subcategories of the categorization framework; comments on Google covered 86% (37/43) of the subcategories, Medicosearch covered 72% (31/43), DocApp covered 60% (26/43), and OkDoc covered 56% (24/43). In total, 2441 distinct issues were identified within the 43 subcategories of the categorization framework; 83.65% (2042/2441) of the issues related to the physician, 6.63% (162/2441) related to the staff, and 9.70% (237/2441) related to the practice. Overall, 95% (41/43) of the subcategories of the categorization framework and 81.60% (1992/2441) of the distinct issues identified were concerning aspects of performance (interpersonal skills of the physician and staff, infrastructure, and organization and management of the practice) that are considered assessable by patients. Overall, 83.0% (705/849) of comments were classified as positive, 2.5% (21/849) as neutral, and 14.5% (123/849) as negative. However, there were significant differences between PRWs, regions, and specialty regarding negative comments: 90.2% (111/123) of negative comments were on Google, 74.7% (92/123) were regarding physicians in Zurich, and 73.2% (90/123) were from specialists.

**Conclusions:**

From the narrative comments analyzed, it can be reported that interpersonal issues make up nearly half of all negative issues identified, and it is recommended that physicians should focus on improving these issues. The current suppression of negative comments by Swiss PRWs is concerning, and there is a need for a consensus-based criterion to be developed to determine which comments should be published publicly. Finally, it would be helpful if Swiss patients are made aware of the current large differences between Swiss PRWs regarding the frequency and nature of ratings to help them determine which PRW will provide them with the most useful information.

## Introduction

Physician rating websites (PRWs) are a sign of the growing digitalization of the patient-health professional relationship, allowing patients to anonymously rate their physicians on the Web as a source of information for others [1-8]. Typically grounded in the assumptions of a theoretical consumer choice model [9], PRWs aim to improve patient welfare through (1) influencing patient decision making by increasing the chance that those patients who obtain information from PRWs will choose better quality physicians and benefit from this [10] and (2) driving quality improvement by identifying aspects of care needing improvement so that changes can be made in the practice [10].

In a recent systematic search of PRWs internationally, 143 different websites from 12 countries were analyzed [11]. The majority of websites were commercially operated by for-profit companies and were registered in the United States and Germany. It was found that 15.3% of these websites only provided the option to give feedback on a predefined quantitative rating scale and 4.2% of websites only provided the option for narrative comments, whereas 76.9% of websites provided the option to give both types of feedback [11].

Narrative comments potentially provide richer insights into patients’ experiences and feelings that cannot be fully captured in predefined quantitative rating scales and are increasingly being examined with content analysis [4,8,12-15], and more recently with machine learning [16-17]. Narrative comments, however, have proved contentious because of concerns that they will be used for *doctorbashing* or defamation [4,18-20]. Although previous research suggests that this concern is largely unfounded [4,8,12-15], physicians’ fear of receiving negative comments on PRWs can have a direct impact on the patient-health professional relationship. For instance, physicians may try to prevent patients from posting negative reviews on PRWs (eg, by asking patients to sign contracts stating they will not make negative comments) and legally challenge negative comments that are posted [3]. Due to the lack of expert knowledge of most patients regarding medicine, there are also concerns about the relevance and significance of their evaluation of physicians’ performance [21]. Although recent research suggests that patients acknowledge their inability to rate physicians’ technical competency [21], an analysis of 3000 narrative comments from the German PRW, jameda, from 2012 found that physicians’ competence was the most frequent issue that patients mentioned in their comments (1874/3000, 62.46%) [4]. It is unclear whether this apparent contradiction, between patients’ agreement about their inability to evaluate physicians’ technical skills and their actual ratings, exists on other PRWs and countries, but the need for more research on this issue has been highlighted [21].

Although the first PRWs in Switzerland, OkDoc and Medicosearch, were established in 2008 at the same time as many international PRWs, there has been a lack of research conducted on PRWs in Switzerland to date. However, a study recently examined, for the first time, the frequency of quantitative ratings and narrative comments on Swiss PRWs [22]. It found that many of the selected physicians could not be identified (42.4% to 87.3%), and few of the identifiable physicians had been rated quantitatively (4.5% to 49.8%) or received narrative comments (4.5% to 31.2%) at least once. Rated physicians also had on average a low number of quantitative ratings (1.47 to 3.74 rating) and narrative comments (1.23 to 3.03 comments) [22]. However, there were significant differences between PRWs, with Google having substantially more quantitative ratings and narrative comments than the 3 dedicated Swiss PRWs in the past two and a half years [22].

Although the content and nature of narrative comments on Swiss PRWs has not been examined to date, the controversial nature of negative comments on Swiss PRWs has received media attention in Switzerland [23-26]. Furthermore, in response to a decision of a federal data commissioner that certain negative comments had to be removed on the PRW OkDoc in a particular case, OkDoc decided to only allow recommendations. OkDoc now explicitly states on its website that any negative comments will be deleted (“Only positive comments recommending your doctor will be accepted. Any negative post will be deleted. Thank you for respecting okdoc's principles!” [author translation]). Although the PRW Medicosearch allows negative comments, it informs the concerned physician before publishing it online so the physician can decide if the negative feedback is activated. However, if the physician refuses, the feedback function is deactivated, also removing the positive comments [23]. This situation potentially raises important implications not only for the frequency of ratings on Swiss PRWs but also for the types of comments that may be available for PRW users. Therefore, this study aimed to examine (1) the types of issues raised in narrative comments published on Swiss PRWs and (2) the evaluation tendencies of the narrative comments. Gaining better understanding regarding this issue may help identify issues that Swiss physicians should focus on to improve patient satisfaction and will also help inform future research and health policy in Switzerland in relation to PRWs.

## Methods

### Sample

A random stratified sample of 966 physicians was generated from the regions of Zürich and Geneva. Zürich is the largest city in Switzerland and is located in north-central Switzerland. Zürich has a total population of 402,762 (12/2016). Geneva is the second largest city in Switzerland and is located in south-western Switzerland. Geneva has a total population of 198,979 (12/2016). The regions of Zürich and Geneva were chosen because of language (German vs French) and a comparable number of total physician (Zürich 3254 physicians and Geneva 2780 physicians) considerations.

In November 2017, all physicians in these regions working in general practice, obstetrics and gynecology, pediatrics, and dermatology and venereology were searched for on the Swiss Medical Association’s medical registry (Ärzteverzeichnis). From each region, a random sample was generated for each specialty based on a 95% confidence level and 5% confidence interval. From Zürich, the random sample consisted of 254 of 747 general practice physicians, 85 of 109 obstetrics and gynecology physicians, 74 of 92 pediatric physicians, and 53 of 61 dermatology and venereology physicians. Therefore, the Zürich sample of 466 physicians represents 46.18% of a total of 1009 physicians. From Geneva, the random sample consisted of 272 of 930 general practice physicians, 86 of 111 obstetrics and gynecology physicians, 96 of 128 pediatric physicians, and 46 of 52 dermatology and venereology physicians. Therefore, the Geneva sample of 500 physicians represents 40.95% of a total of 1221 physicians (see Table 1).

**Table 1:** Physician samples per region.

Specialty	Zurich		Geneva		Total	
	Total physicians found, N	Physicians selected for sample, n (%)	Total physicians found, N	Physicians selected for sample, n (%)	Total physicians found, N	Physicians selected for sample, n (%)
General practitioners	747	254 (34.0)	930	272 (29.2)	1677	526 (31.36)
Obstetrics and gynecology	109	85 (77.9)	111	86 (77.5)	220	171 (77.7)
Pediatrics	92	74 (80.4)	128	96 (75.0)	220	170 (77.3)
Dermatology and venereology	61	53 (86.8)	52	46 (88.5)	113	99 (87.6)
Total	1009	466 (46.18)	1221	500 (40.95)	2230	966 (43.32)

### Data Collection

To identify PRWs on which patients can rate and review physicians in Switzerland, a systematic online search was conducted in June 2016 from a patient’s perspective [22]. A website was included if it allowed users to view quantitative ratings and/or narrative comments about Swiss physicians in a structured manner without having to open an account or log onto the website. Websites that were not dedicated to Swiss physicians were excluded. A total of 3 PRWs were included: OkDoc, DocApp, and Medicosearch. In addition, Google itself allows users to rate and comment on physicians via Google reviews. Furthermore, although the health care information portal doktor does not provide the option for ratings, it links to Google reviews. Google was therefore also included in the study, and as far as this author is aware, this is the first time Google has been included in a study examining physician ratings internationally. The selected physicians were therefore searched for on a total of 4 websites: OkDoc, DocApp, Medicosearch, and Google. On each website, every selected physician was searched for between November 2017 and July 2018 and any narrative comments were recorded.

### Data Analysis

The content of each narrative comment was analyzed and classified by the author according to a theoretical categorization framework of physician-, staff-, and practice-related issues. The categorization framework from Emmert et al was initially used [4], with modifications being made where necessary. This included removing categories that were not identified in the comments, adding categories that were identified but were not adequately covered by the previous framework, and separating categories (eg, friendliness and caring attitude) that were discussed in comments as distinct issues. Narrative comments were also classified as positive, neutral, and negative, overall. If a comment included both positive and negative aspects and no clear tendency could be determined, the comment was categorized as neutral. Narrative comments were analyzed in their original language. Descriptive statistics included means and standard deviations for continuous variables and percentages for categorical variables. To analyze whether differences exist between different groups, chi-squared tests were used for categorical data and *t* tests, for continuously distributed data. In relation to chi-squared tests with the 4 PRWs, posthoc tests using Bonferroni correction were conducted for the significant results to identify which PRW differed from the others. All analyses were performed with a significance level alpha set to .05 and 2-tailed tests, using Statistical Package for the Social Sciences (SPSS version 24 for Windows, IBM Corporation).

## Results

### Nature of Comments

The selected physicians in the sample had a total of 849 comments. Table 2 shows the breakdown of the number of comments by region, specialty, and gender. Overall, comments were significantly more likely to be regarding physicians in Zurich (668/849, 78.7%), specialists (545/849, 64%), and male physicians (477/849, 56.2%). However, there were important differences between PRWs. Although specialists (373/520, 71.7%) had significantly more comments on Google (χ2_1_=98.2; *P*<.001), there were no significant differences between general practitioners and specialists on OkDoc, DocApp, or Medicosearch. Furthermore, although male physicians had more comments on okdoc (24/38, 63%) and significantly more (χ2_1_=33.5; *P*<.001) on Google (326/520, 62.7%), female physicians had more comments on DocApp (30/57, 52%) and significantly more (χ2_1_=4.9; *P*<.03) on Medicosearch (134/234, 57.3%).

The 849 comments had a mean length of 253.5 characters (SD 298), ranging from 15 to 3258 characters. There was a significant difference in the mean character length of the following groups:

- Positive comments (mean 222, SD 224) and negative comments (mean 436, SD 533); *t*_130_=−4.4; *P*<.001.
- Physicians from Zurich (mean 231, SD 242) and physicians from Geneva (mean 335, SD 439); *t*_210_=−3.1; *P*=.003.
- General practitioners (mean 193, SD 167) and specialists (mean 288, SD 347); *t*_830_=−5.4; *P*<.001.
- Okdoc (mean 154, SD 126), DocApp (mean 296, SD 202), Medicosearch (mean 174, SD 146), and Google (mean 292, SD 354); *F*_3_=10.4; *P*<.001.

However, there was no significant difference in the mean character length of male physicians (mean 256, SD 291) and female physicians (mean 250, SD 307); *t*_847_=0.3; *P*=.77.

### Categorization of Issues

The analysis of the 849 comments identified 43 subcategories addressing the physician (n=21), the staff (n=8), and the practice (n=14; see Textbox 1).

None of the PRWs’ comments covered all 43 subcategories of the categorization framework (see Table 3); comments on Google covered 86% (37/43) of the subcategories, Medicosearch covered 72% (31/43), DocApp covered 60% (26/43), and OkDoc covered 55% (24/43).

**Table 2:** Physicians with comments.

Physician characteristics		OkDoc	DocApp	Medicosearch	Google	Total
**Physician region**						
	Zurich, n/N (%)	20/38 (52.6)	56/57 (98.2)	206/234 (88)	386/520 (74.2)	668/849 (78.7)
	Geneva, n/N (%)	18/38 (47.4)	1/57 (1.8)	28/234 (12)	134/520 (25.8)	181/849 (21.3)
	Chi-squared test (*df*)	0.11 (1)	53.1 (*1*)	135.4 (*1*)	122.1 (*1*)	46.7 (*3*)
	*P* value	.75	<.001	<.001	<.001	<.001
**Physician specialty**						
	General practitioners, n/N (%)	23/38 (60.5)	28/57 (49.1)	108/234 (46.2)	147/520 (28.3)	306/849 (26)
	Specialists, n/N (%)	15/38 (39.5)	29/57 (50.9)	126/234 (53.8)	373/520 (71.7)	543/849 (64)
	Chi-squared test (*df*)	1.6 (*1*)	0.02 (*1*)	1.4 (*1*)	98.2 (*1*)	38.1(*3*)
	*P* value	.19	.90	.24	<.001	<.001
**Physician gender**						
	Male, n/N (%)	24/38 (63.2)	27/57 (47.4)	100/234 (42.7)	326/520 (62.7)	477/849 (56.2)
	Female, n/N (%)	14/38 (36.8)	30/57 (52.6)	134/234 (57.3)	194/520 (37.3)	372/849 (43.8)
	Chi-squared test (*df*)	2.6 (*1*)	0.20 (*1*)	4.9 (*1*)	33.5 (*1*)	28.7 (*3*)
	*P* value	.11	.70	.03	<.001	<.001

**Categorization framework.**

Physician (n=21)

- Overall assessment; Competence; Communication; Recommendation; Friendliness; Caring attitude; Satisfaction with treatment; Professionalism; Time spent with patient; Trust; Treatment cost/billing; Being taken seriously; Cooperation with medical specialists; Alternative medicine; Patient involvement; Telephone availability; Individualized service; House visits; Available outside normal hours; Privacy; Health insurance differentiation

Staff (n=8)

- Friendliness; Service/assistance; Overall assessment; Professionalism; Communication; Availability by telephone; Recommendation; Time spent with patient

Practice (n=14)

- Atmosphere; Waiting time within practice; Ability to get appointment; Overall assessment; Location; Organization; Equipment; Online appointment; Recommendation; Parking space; Consultation hours; Waiting room entertainment; Availability by telephone; Barrier-free access

**Table 3:** Subcategories covered by physician rating websites’ comments.

Subcategories	OkDoc	DocApp	Medicosearch	Google
Physician (N=21), n (%)	16 (76)	14 (66)	17 (80)	18 (85)
Staff (N=8), n (%)	3 (37)	4 (50)	5 (62)	6 (75)
Practice (N=14), n (%)	5 (35)	8 (57)	9 (64)	13 (92)
Total (N=43), n (%)	24 (55)	26 (60)	31 (72)	37 (86)

In total, 2441 distinct issues were identified within the 43 subcategories of the categorization framework; 83.65% (2042/2441) of the issues were related to the physician, 6.63% (162/2441) related to the staff, and 9.70% (237/2441) related to the practice (see Table 4). Overall, the 2 most frequently issues mentioned were the overall assessment of the physician (300/849, 35.3%) and the physician’s competence (300/849, 35.3%); the vast majority of these comments were positive (92.7% and 94.7%, respectively). Other frequently mentioned issues regarding the physician included 27.3% (232/849) of comments referred to the physician’s communication (84.9% positive); 26.5% (225/849) recommended the physician (86.2% positive); 25.3% (215/849) referred to the physician’s friendliness (88.8% positive); 22.6% (192/849) referred to the physician’s caring attitude (87.5% positive); 17.6% (149/849) referred to satisfaction with treatment (79.2% positive); 15.2% (129/849) referred to the physician’s professionalism (76.7% positive); 12.6% (107/849) referred to time spent with the patient (87.9% positive); and 9.7% (82/849) referred to the physician’s trustworthiness (89% positive). In relation to staff issues, the most frequently mentioned issue was regarding the staff’s friendliness (92/849, 10.8%), 84.8% of which were positive. Concerning practice issues, frequently mentioned issues included 6.9% (59/849) of comments that mentioned the atmosphere of the practice (91.5% positive), 6.8% (58/849) the waiting time within the practice (72.4% positive), and 4.6% (39/849) the ability to get an appointment (79.5% positive). Negative comments most frequently referred to treatment cost or billing (32/43, 74%), communication with the staff (7/13, 53%), the staff’s professionalism (4/15, 26%), waiting time within practice (12/58, 20%), ability to get an appointment (8/39, 20%), the physician’s professionalism (26/129, 20.2%), and satisfaction with treatment (27/149, 18.1%).

However, there were some significant differences between PRWs, regions, specialties, and gender (see Multimedia Appendices 1-4 for full results). Regarding the PRWs, there were significant differences between comments on Google and the 3 dedicated PRWs in a number of subcategories. For instance, there were significant differences between PRWs in relation to comments mentioning the physician’s competency (χ2_3_=11.4; *P*=.01; V=0.12). Posthoc tests using Bonferroni correction revealed that comments on Google (161/520, 31.0%) mentioned the physician’s competence significantly less than the overall sample. There were also significant differences between PRWs regarding satisfaction with treatment (χ2_3_=16.9; *P*<.001; V=0.14). Posthoc tests using Bonferroni correction revealed that comments on Google (111/520; 21.3%) referred to satisfaction with treatment significantly more than the overall sample. Furthermore, 97% (42/43) of the references to treatment cost or billing issues were made in comments from Google.

There were significant differences between comments regarding physicians from Zurich and Geneva in a number of subcategories. For instance, comments regarding physicians from Zurich mentioned the physician’s competence (263/668, 39.3%) significantly more often (χ2_1_=22.3; *P*<.001) than comments from physicians from Geneva (37/181, 20.4%). However, physicians from Geneva had a higher percentage of comments referring to the physician’s communication (60/181, 33.1% vs 172/668, 25.7%), the physician’s caring attitude (50/181, 27.6% vs 142/668, 21.2%), the physician’s professionalism (39/181, 21.5% vs 90/668, 13.4%), and trust in the physician (24/181, 13.2% vs 58/668, 8.6%). Comments regarding specialists significantly more often recommended the physician (χ2_1_=8.6; *P*=.004), the physician’s caring attitude (χ2_1_=8.6; *P*=.004), satisfaction with treatment (χ2_1_=9.9; *P*=.002), treatment cost and billing (χ2_1_=9.6; *P*=.002), staff friendliness (χ2_1_=12.2; *P*<.001), and practice atmosphere (χ2_1_=6.8; *P*=.01). Comments regarding male physicians (102/477, 21.3%) were significantly more (χ2_1_=11.1; *P*=.001) likely to refer to satisfaction with treatment than comments about female physicians (47/372, 12.6%). However, comments regarding female physicians (22/372, 5.9%) were significantly more (χ2_1_=11.0; *P*=.001) likely to mention that the patient felt like they had been taken seriously than comments about male physicians (8/477, 1.6%).

### Evaluation Results

Overall, 83.0% (705/849) of comments were classified as positive, 2.5% (21/849) as neutral, and 14.5% (123/849) as negative (see Table 5). However, there were significant differences between PRWs, regions, and specialty regarding negative comments: 90.2% (111/123) of negative comments were on Google (χ2_2_=180.1; *P*<.001), 74.7% (92/123) were regarding physicians in Zurich (χ2_1_=30.3; *P*<.001), and 73.2% (90/123) were regarding specialists (χ2_1_=26.4; *P*<.001). There was no significant difference (χ2_1_=2.4; *P*=.13) between males (70/123, 56.9%) and females (53/123, 43.1%) regarding negative comments.

**Table 4:** Categorization of issues.

Issue		Total (N=849), n (%)	Evaluation		
			Positive, n (%)	Neutral, n (%)	Negative, n (%)
**Physician**					
	Overall assessment	300 (35.3)	278 (92.7)	7 (2.3)	15 (5.0)
	Competence	300 (35.3)	284 (94.7)	5 (1.7)	11 (3.7)
	Communication	232 (27.3)	197 (84.9)	2 (0.9)	33 (14.2)
	Recommendation	225 (26.5)	194 (86.2)	0 (0.0)	31 (13.8)
	Friendliness	215 (25.3)	191 (88.8)	5 (2.3)	19 (8.8)
	Caring attitude	192 (22.6)	168 (87.5)	3 (1.6)	21 (10.9)
	Satisfaction with treatment	149 (17.6)	118 (79.2)	4 (2.7)	27 (18.1)
	Professionalism	129 (15.2)	99 (76.7)	4 (3.1)	26 (20.2)
	Time spent with patient	107 (12.6)	94 (87.9)	2 (1.9)	11 (10.3)
	Trust	82 (9.7)	73 (89)	0 (0)	9 (11)
	Treatment cost/billing	43 (5.1)	10 (23)	1 (2)	32 (74)
	Being taken seriously	30 (3.5)	25 (83)	0 (0)	5 (16)
	Cooperation with medical specialists	11 (1.3)	11 (100)	0 (0)	0 (0)
	Alternative medicine	5 (0.6)	5 (100)	0 (0)	0 (0)
	Patient involvement	5 (0.6)	5 (100)	0 (0)	0 (0)
	Telephone availability	5 (0.6)	4 (80)	0 (0)	1 (20)
	Individualized service	4 (0.5)	4 (100)	0 (0)	0 (0)
	House visits	3 (0.4)	3 (100)	0 (0)	0 (0)
	Available outside normal hours	2 (0.2)	2 (100)	0 (0)	0 (0)
	Privacy	2 (0.2)	2 (100)	0 (0)	0 (0)
	Health insurance differentiation	1 (0.1)	0 (0)	0 (0)	1 (100)
**Staff**					
	Friendliness	92 (10.8)	78 (84)	6 (6)	8 (8)
	Service/assistance	19 (2.2)	17 (89)	0 (0)	2 (10)
	Overall assessment	18 (2.1)	16 (88)	1 (5)	1 (5)
	Professionalism	15 (1.8)	10 (66)	1 (6)	4 (26)
	Communication	13 (1.5)	5 (38)	1 (7)	7 (53)
	Availability by telephone	3 (0.4)	3 (100)	0 (0)	0 (0)
	Recommendation	1 (0.1)	1 (100)	0 (0)	0 (0)
	Time spent with patient	1 (0.1)	1 (100)	0 (0)	0 (0)
**Practice**					
	Atmosphere	59 (6.9)	54 (91)	3 (5)	2 (3)
	Waiting time within practice	58 (6.8)	42 (72)	4 (6)	12 (20)
	Ability to get appointment	39 (4.6)	31 (79)	0 (0)	8 (20)
	Overall assessment	22 (2.6)	20 (90)	1 (4)	1 (4)
	Location	15 (1.8)	13 (86)	0 (0)	2 (13)
	Organization	13 (1.5)	10 (76)	1 (7)	2 (15)
	Equipment	9 (1.1)	8 (88)	0 (0)	1 (11)
	Online appointment	5 (0.6)	5 (100)	0 (0)	0 (0)
	Recommendation	5 (0.6)	5 (100)	0 (0)	0 (0)
	Parking space	5 (0.6)	5 (100)	0 (0)	0 (0)
	Consultation hours	2 (0.2)	2 (100)	0 (0)	0 (0)
	Waiting room entertainment	2 (0.2)	2 (100)	0 (0)	0 (0)
	Availability by telephone	2 (0.2)	1 (50)	0 (0)	1 (50)
	Barrier free access	1 (0.1)	0 (0)	1 (100)	0 (0)

**Table 5:** Evaluation results.

Region		OkDoc, n/N (%)	DocApp, n/N (%)	Medicosearch, n/N (%)	Google, n/N (%)	Total, n/N (%)
**Zurich**						
	Positive	19/20 (95)	54/56 (96)	192/206 (93.2)	293/386 (74.9)	558/668 (83.5)
	Neutral	1/20 (5)	0/56 (0)	5/206 (2.4)	12/386 (3.1)	18/668 (2.7)
	Negative	0/20 (0)	2/56 (3)	9/206 (4.4)	81/386 (21)	92/668 (13.8)
**Geneva**						
	Positive	18/18 (100)	1/1 (100)	27/28 (96)	101/134 (75.4)	147/181 (81.2)
	Neutral	0/18 (0)	0/1 (0)	0/28 (0)	3/134 (2.2)	3/181 (1.7)
	Negative	0/18 (0)	0/1 (0)	1/28 (3)	30/134 (22.4)	31/181 (17.1)
**Total**						
	Positive	37/38 (97)	55/57 (96)	219/234 (93.6)	394/520 (75.8)	705/849 (83)
	Neutral	1/38 (2)	0/57 (0)	5/234 (2.1)	15/520 (2.9)	21/849 (2.5)
	Negative	0/38 (0)	2/57 (3)	10/234 (4.3)	111/520 (21.3)	123/849 (14.5)

## Discussion

As far as this author is aware, this is the first study to examine the content and nature of narrative comments on Swiss PRWs and has resulted in a number of key findings: (1) the vast majority of issues mentioned were concerning aspects of performance (interpersonal skills of physician and staff, infrastructure, and organization and management of practice) that are considered assessable by patients; (2) overall, the vast majority of comments were positive; and (3) there were significant differences between comments on Google and comments on the 3 dedicated PRWs.

### Content of Comments

The 5 most frequently mentioned issues identified from the narratives comments were (1) the overall assessment of the physician (300/849, 35.3%) and the physician’s competence (300/849, 35.3%); (2) the physician’s communication (232/849, 27.3%); (3) recommending the physician (225/849, 26.5%); (4) the physician’s friendliness (215/849, 25.3%); and 5) the physician’s caring attitude (192/849, 22.6%). In contrast, the top 5 mentioned issues identified by Emmert et al’s analysis of 3000 narrative comments from the German PRW, jameda, from 2012 were as follows: (1) the physician’s competence (1874/3000, 62.46%); (2) the physician’s friendliness and caring attitude (1148/3000, 38.26%); (3) the time the physician spent with the patient (987/3000, 32.90%); (4) the friendliness of the staff (667/3000, 22.23%); and (5) the information and advice from the physician (630/3000, 21.00%) [4].

Although both studies found that narrative comments most frequently mentioned the physician’s competence, it should be noted that while this study kept the issues of *the physician’s friendliness* and *the physician’s caring attitude* separate, Emmert et al combined the 2 issues [4]. If this study also combined these 2 issues, the physician’s friendliness and caring attitude would become the most frequently mentioned issue (407/849, 47.9%). Indeed, it is important to recognize that 95% (41/43) of the subcategories of the categorization framework and 81.60% (1992/2441) of the distinct issues identified were concerning aspects of performance (interpersonal skills of physician and staff, infrastructure, and organization and management of practice) that are considered to be assessable by patients [21]. Although a number of narrative comments also mentioned the physician’s competency (300/849, 35.3%), the proportion of comments that mentioned this issue were substantially lower than that reported by Emmert et al (62.5%) [4].

Unsolicited critical comments on PRWs can be seen as a type of complaint, which can offer a *window of opportunity* to improve health services [27]. Indeed, one of the aims of PRWs is to drive quality improvement by identifying aspects of care needing improvement so that changes can be made in practice [10]. Overall, 123 comments were classified as negative. Within these negative comments, 293 distinct issues were identified. Nearly half of all negative issues (132/293, 45.1%) concerned interpersonal issues: the physician’s communication (n=33), the physician’s friendliness (n=19), the physician’s caring attitude (n=21), the physician’s professionalism (n=26), the physician’s trustworthiness (n=9), being taken seriously by the physician (n=5), the friendliness of the staff (n=8), the professionalism of staff (n=4), and staff communication (n=7). Given these interpersonal issues make up nearly half of all negative issues, and that improving these issues will potentially also improve the patient’s overall assessment and recommendation of physicians (46/293, 15.7% of negative issues), it is recommended that physicians should focus on improving interpersonal interactions with patients. However, the health care setting can be a very stressful and emotionally draining environment because of external (including workload, exposure to patient suffering, time pressures, documentation requirement, and financial issues) and internal (including personality characteristics and poor emotional regulation) factors [28]. This can lead to stress, dissatisfaction, increased cynicisms, burnout, and compassion fatigue among health care professionals and the staff [28,29]. In recent decades, the Swiss health care system has experienced a number of changes that have caused greater economic constraints, increased administrative workload, and decreased professional autonomy [30]. A study published in 2010 found that burnout levels among Swiss physicians had increased throughout the country over the last decade [30]. The increased burnout levels among Swiss physicians may be contributing to the suboptimal interpersonal issues reported in the narrative comments. Although there are strategies that individual physicians can use to improve their interpersonal skills [28], to really address this issue, whole-system approaches may be required to improve the well-being of physicians [29].

### Nature of Comments

The analysis of the 849 narrative comments on Swiss PRWs reveals that 83% (705/849) of all comments were positive, 2.5% (21/849) were neutral, and 14.5% (123/849) were negative. This finding is very similar to the previous analysis of narrative comments on PRWs in other countries [4,8,12-15]. For example, Emmert et al’s analysis of 3000 narrative comments from the German PRW, jameda, from 2012 found that 80% of all comments were positive, 4% were neutral, and 16% were negative [4]. Although this finding suggests that the users of Swiss PRWs are mostly satisfied with their physicians, the veracity of the level of satisfaction must be called into question given the explicit practice of the dedicated PRWs of not allowing negative comments or removing them if physicians do not want them published. On OkDoc, 0 of the 38 comments were negative; on DocApp, 2 of the 57 comments were negative; and on Medicosearch, 10 of the 234 comments were negative. Although Google had 90.2% (111/123) of negative comments, the author has become aware that some negative comments that were online during data collection have since been removed. It is, therefore, unclear how many negative comments are being supressed on Swiss PRWs. However, the current suppression of negative comments by Swiss PRWs is concerning and goes against their overall aim of achieving more transparency. There are, no doubt, challenges in finding the correct balance between protecting physicians from harm and promoting the health literacy benefits for patients. However, a blanket ban on negative comments or removing comments simply because the physician in question does not like a particular comment seems inappropriate and is leading to a biased and inaccurate picture of patients’ experiences and satisfaction. There is a need for a consensus-based criterion that applies to all Swiss PRWs for determining which comments are to be and not to be published publically and which are clearly publicized so users of PRWs are aware of it. Indeed, a recent qualitative study conducted with a random sample of residents of 4 North German cities reported that a lack of rating guidance in terms of allowable content was a disincentive for rating a physician on a PRW [31]. It is also likely that the removal of a comment on the whims of a PRW operator is a disincentive for users to give further physician ratings in the future. Such criteria should also address how comments that PRWs suspect to be *fake reviews* should be handled, as there is some indication that physicians or practice staff sometimes pose as patients on PRWs to post either positive comments about themselves or negative comments about competitors [32].

### Google

As far as this author is aware, this is the first time Google has been included in a project examining physician ratings internationally. It has already been reported that Google had the highest average number of quantitative ratings (3.74 ratings) and narrative comment (3.03 comments) ratings per identifiable physician [22]. This analysis of the content and nature of the narrative comments on Swiss PRWs reveals that the comments on Google are also far richer than the comments on the other Swiss PRWs; comments on Google covered the most subcategories of the categorization framework (37/43, 86%) and also had the majority of negative comments (111/123, 90.2%). It, therefore, appears that Google has not only become the most used website in Switzerland for physician ratings in recent years but is also potentially the most useful. It would be helpful if Swiss patients are made aware of the current large differences between Swiss PRWs regarding the frequency and nature of ratings to help them determine which PRW will provide them with the most useful information. However, future updates would be helpful to assess whether Google, given its general market dominance, will take an even bigger share of the PRW ratings away from the dedicated PRW competitors, or whether the dedicated PRWs will be able to increase the quantity and quality of ratings. Indeed, Medicosearch has already started to shift its business strategy toward online appointments, something that Google does not currently offer, which may allow them to gain a bigger market share and increase the number of ratings. However, it may be necessary for OkDoc to reflect on whether their continued existence in the Swiss PRW market is providing value or is in fact causing harm. It has already been reported that OkDoc had the lowest average number of quantitative ratings (1.47 ratings) and narrative comment (1.23 comments) ratings per identifiable physician, and it only had one comment posted for all 966 physicians in the sample during the last five and a half years (2012-2018) [22]. This analysis of the content and nature of the narrative comments has also found that OkDoc covered the least amount of subcategories of the categorization framework (24/43, 55%) and that it does not have any negative comments.

### Limitations

This study has a number of limitations that should be taken into account when interpreting the results. First, although a systematic online search of Swiss PRWs was conducted, there may be other types of websites that allow Swiss physicians to be rated that were not included in this study. This is a fast-moving area, and it does appear that there are some websites that have started allowing ratings or making ratings publically available after this project had commenced (eg, deindoktor and doctena), which should be added to any future studies examining PRWs in Switzerland. Second, only German search terms were used for the systematic online search of Swiss PRWs. Although the author is confident that no important Swiss PRWs were missed at the time of developing and conducting the project, it would be preferable if French and Italian search terms are also included in future research in Switzerland to ensure that no PRWs are being missed. Third, the sample was only taken from 2 regions in Switzerland, which may limit the generalizability of the results. Although the study used a representative random sample from a German-speaking and French-speaking region of Switzerland with a comparable number of physicians, given the significant differences found between the 2 regions, it would be helpful for further research to include other regions to examine whether these differences are found between other German- and French-speaking regions and in the Italian-speaking region of Ticino. Fourth, a distinction was only made between general practitioners and specialists, and there may be further differences between the different specialties. Finally, the sociodemographic information of the rating patients is unknown and may not be representative of Swiss patients in general.

## Appendix

Multimedia Appendix 1 Categorization of issues by physician rating websites.

Multimedia Appendix 2 Categorization of issues by regions.

Multimedia Appendix 3 Categorization of issues by specialties.

Multimedia Appendix 4 Categorization of issues by gender.

## Abbreviations

PRW: physician rating website
