# Supplementary material for: The Content and Nature of Narrative Comments on Swiss Physician Rating Websites: Analysis of 849 Comments
Source: J Med Internet Res. 2019 Sep 30;21(9):e14336. doi: 10.2196/14336 (PMC6792026; doi:10.2196/14336)
Supplement: Multimedia Appendix 1 [file jmir_v21i9e14336_app1.pdf]

Multimedia Appendix 1. Categorisation of issues by physician rating websites (PRWs)

Issue	PRW (%)	Chi-squared-test	Evaluation % (+/=/-)
**Physician (N=2042)**			
Overall assessment<br>(n=300)	okdoc: 15/38 (39.5)<br>docapp: 18/57 (31.6)<br>medicosearch: 83/234 (35.5)<br>google: 184/520 (35.4)	$\chi^2_{(3)}$=.64,<br>*P*=.89	15(100) / 0(0) / 0(0)<br>18(100) / 0(0) / 0(0)<br>80(96.4) / 2(2.4) / 1(1.2)<br>165(89.7) / 5(2.7) / 14(7.6)
Competence<br>(n=300)	okdoc: 17/38 (44.7)<br>docapp: 24/57 (42.1)<br>medicosearch: 98/234 (41.9)<br>google: 161/520 (31.0)	**$\chi^2_{(3)}$=11.4,**<br>***P*=.01, V=.12**	16(94.1) / 1(5.9) / 0(0)<br>24(100) / 0(0) / 0(0)<br>95(96.9) / 1(1.0) / 2(2.0)<br>149(92.5) / 3(1.9) / 9(5.6)
Communication<br>(n=232)	okdoc: 12/38 (31.6)<br>docapp: 18/57 (31.6)<br>medicosearch: 69/234 (29.5)<br>google: 133/520 (25.6)	$\chi^2_{(3)}$=2.2,<br>*P*=.53	12(100) / 0(0) / 0(0)<br>18(100) / 0(0) / 0(0)<br>64(92.8) / 2(2.9) / 3(4.3)<br>103(88.4) / 0(0) / 30(22.6)
Recommendation<br>(n=225)	okdoc: 7/38 (18.4)<br>docapp: 22/57 (38.6)<br>medicosearch: 42/234 (17.9)<br>google: 154/520 (29.6)	**$\chi^2_{(3)}$=16.9,**<br>***P*<.001, V=.14**	7(100) / 0(0) / 0(0)<br>22(100) / 0(0) / 0(0)<br>40(95.2) / 0(0) / 2(4.8)<br>125(81.2) / 0(0) / 29(18.8)
Friendliness<br>(n=215)	okdoc: 6/38 (15.8)<br>docapp: 21/57 (36.8)<br>medicosearch: 62/234 (26.5)<br>google: 126/520 (24.2)	$\chi^2_{(3)}$=6.3,<br>*P*=.10	6(100) / 0(0) / 0(0)<br>19(90.5) / 0(0) / 2(9.5)<br>59(95.2) / 1(1.6) / 2(3.2)<br>107(84.9) / 4(3.2) / 15(11.9)

Caring attitude<br><br>(n=192)	okdoc: 10/38 (26.3)<br><br>docapp: 18/57 (31.6)<br><br>medicosearch: 55/234 (23.5)<br><br>google: 109/520 (21.0)	$\chi^2_{(3)}$=3.8,<br><br>*P*=.28	10(100) / 0(0) / 0(0)<br><br>17(94.4) / 0(0) / 1(5.6)<br><br>51(92.7) / 2(3.6) / 2(3.6)<br><br>90(82.6) / 1(0.9) / 18(16.5)
Satisfaction with treatment<br><br>(n=149)	okdoc: 1/38 (2.6)<br><br>docapp: 10/57 (17.5)<br><br>medicosearch: 27/234 (11.5)<br><br>google: 111/520 (21.3)	**$\chi^2_{(3)}$=16.9,**<br><br>***P*<.001, V=.14**	1(100) / 0(0) / 0(0)<br><br>10(100) / 0(0) / 0(0)<br><br>24(88.9) / 2(7.4) / 1(3.7)<br><br>83(74.8) / 2(1.8) / 26(23.4)
Professionalism<br><br>(n=129)	okdoc: 3/38 (7.9)<br><br>docapp: 11/57 (19.3)<br><br>medicosearch: 19/234 (8.1)<br><br>google: 96/520 (18.5)	**$\chi^2_{(3)}$=15.7,**<br><br>***P*<.001, V=.14**	3(100) / 0(0) / 0(0)<br><br>11(100) / 0(0) / 0(0)<br><br>17(89.5) / 1(5.3) / 1(5.3)<br><br>68(70.8) / 3(3.1) / 25(26.0)
Time spent with patient<br><br>(n=107)	okdoc: 6/38 (15.8)<br><br>docapp: 5/57 (8.8)<br><br>medicosearch: 42/234 (17.9)<br><br>google: 54/520 (10.4)	**$\chi^2_{(3)}$=9.5,**<br><br>***P*=.02, V=.11**	6(100) / 0(0) / 0(0)<br><br>5(100) / 0(0) / 0(0)<br><br>39(92.9) / 1(2.4) / 2(4.8)<br><br>44(81.5) / 1(1.9) / 9(16.7)
Trust<br><br>(n=82)	okdoc: 5/38 (13.2)<br><br>docapp: 2/57 (3.5)<br><br>medicosearch: 55/234 (23.5)<br><br>google: 20/520 (3.8)	**$\chi^2_{(3)}$=74.5,**<br><br>***P*<.001, V=.30**	5(100) / 0(0) / 0(0)<br><br>2(100) / 0(0) / 0(0)<br><br>54(98.2) / 0(0) / 1(1.8)<br><br>12(60) / 0(0) / 8(40)
Treatment cost/billing<br><br>(n=43)	okdoc: 0/38 (0.0)<br><br>docapp: 0/57 (0.0)<br><br>medicosearch: 1/234 (0.4)<br><br>google: 42/520 (8.1)	**$\chi^2_{(3)}$=25.4,**<br><br>***P*<.001, V=.17**	0(0) / 0(0) / 0(0)<br><br>0(0) / 0(0) / 0(0)<br><br>1(100) / 0(0) / 0(0)<br><br>9(21.4) / 1(2.4) / 32(76.2)
Being taken seriously	okdoc: 0/38 (0.0)	**$\chi^2_{(3)}$=28.3,**	0(0) / 0(0) / 0(0)

(n=30)	docapp: 1/57 (1.8)<br>medicosearch: 21/234 (9.0)<br>google: 8/520 (1.5)	***P*<.001, V=.18**	1(100) / 0(0) / 0(0)<br>18(85.7) / 0(0) / 3(14.3)<br>6(75) / 0(0) / 0(25)
Cooperation with medical specialists<br>(n=11)	okdoc: 1/38 (2.6)<br>docapp: 2/57 (3.5)<br>medicosearch: 4/234 (1.7)<br>google: 4/520 (0.8)	$\chi^2_{(3)}$=4.2,<br>*P*=.25	1(100) / 0(0) / 0(0)<br>2(100) / 0(0) / 0(0)<br>4(100) / 0(0) / 0(0)<br>4(100) / 0(0) / 0(0)
Alternative medicine<br>(n=5)	okdoc: 1/38 (2.6)<br>docapp: 1/57 (1.8)<br>medicosearch: 1/234 (0.4)<br>google: 2/520 (0.4)	$\chi^2_{(3)}$=4.5,<br>*P*=.21	1(100) / 0(0) / 0(0)<br>1(100) / 0(0) / 0(0)<br>1(100) / 0(0) / 0(0)<br>2(100) / 0(0) / 0(0)
Patient involvement<br>(n=5)	okdoc: 2/38 (5.3)<br>docapp: 1/57 (1.8)<br>medicosearch: 2/234 (0.9)<br>google: 0/520 (0.0)	**$\chi^2_{(3)}$=18.9,**<br>***P*<.001, V=.15**	2(100) / 0(0) / 0(0)<br>1(100) / 0(0) / 0(0)<br>2(100) / 0(0) / 0(0)<br>0(0) / 0(0) / 0(0)
Telephone availability<br>(n=5)	okdoc: 1/38 (2.6)<br>docapp: 0/57 (0.0)<br>medicosearch: 1/234 (0.4)<br>google: 3/520 (0.6)	$\chi^2_{(3)}$=3.2,<br>*P*=.37	1(100) / 0(0) / 0(0)<br>0(0) / 0(0) / 0(0)<br>1(100) / 0(0) / 0(0)<br>2(66.7) / 0(0) / 1(33.3)
Individualised service<br>(n=4)	okdoc: 0/38 (0.0)<br>docapp: 0/57 (0.0)<br>medicosearch: 0/234 (0.0)<br>google: 4/520 (0.8)	$\chi^2_{(3)}$=2.5,<br>*P*=.47	0(0) / 0(0) / 0(0)<br>0(0) / 0(0) / 0(0)<br>0(0) / 0(0) / 0(0)<br>4(100) / 0(0) / 0(0)
House visits<br>(n=3)	okdoc: 2/38 (5.3)<br>docapp: 0/57 (0.0)	**$\chi^2_{(3)}$=28.1,**<br>***P*<.001, V=.18**	2(100) / 0(0) / 0(0)<br>0(0) / 0(0) / 0(0)

	medicosearch: 1/234 (0.4)<br>google: 0/520 (0.0)		1(100) / 0(0) / 0(0)<br>0(0) / 0(0) / 0(0)
Available outside normal hours<br>(n=2)	okdoc: 0/38 (0.0)<br>docapp: 0/57 (0.0)<br>medicosearch: 0/234 (0.0)<br>google: 2/520 (0.4)	$\chi^2_{(3)}$=1.3,<br>*P*=.74	0(0) / 0(0) / 0(0)<br>0(0) / 0(0) / 0(0)<br>0(0) / 0(0) / 0(0)<br>2(100) / 0(0) / 0(0)
Privacy<br>(n=2)	okdoc: 2/38 (5.3)<br>docapp: 0/57 (0.0)<br>medicosearch: 0/234 (0.0)<br>google: 0/520 (0.0)	**$\chi^2_{(3)}$=43.0,**<br>***P*<.001, V=.22**	2(100) / 0(0) / 0(0)<br>0(0) / 0(0) / 0(0)<br>0(0) / 0(0) / 0(0)<br>0(0) / 0(0) / 0(0)
Health insurance differentiation<br>(n=1)	okdoc: 0/38 (0.0)<br>docapp: 0/57 (0.0)<br>medicosearch: 0/234 (0.0)<br>google: 1/520 (0.2)	$\chi^2_{(3)}$=.63,<br>*P*=.90	0(0) / 0(0) / 0(0)<br>0(0) / 0(0) / 0(0)<br>0(0) / 0(0) / 0(0)<br>0(0) / 0(0) / 1(100)
**Staff (N=162)**			
Friendliness<br>(n=92)	okdoc: 4/38 (10.5)<br>docapp: 8/57 (14.0)<br>medicosearch: 9/234 (3.8)<br>google: 71/520 (13.7)	**$\chi^2_{(3)}$=16.7,**<br>***P*=.001, V=.14**	4(100) / 0(0) / 0(0)<br>7(87.5) / 0(0) / 1(12.5)<br>6(66.7) / 2(22.2) / 1(11.1)<br>61(85.9) / 4(5.6) / 6(8.5)
Service/assistance<br>(n=19)	okdoc: 0/38 (0.0)<br>docapp: 0/57 (0.0)<br>medicosearch: 0/234 (0.0)<br>google: 19/520 (3.7)	**$\chi^2_{(3)}$=12.3,**<br>***P*=.006, V=.12**	0(0) / 0(0) / 0(0)<br>0(0) / 0(0) / 0(0)<br>0(0) / 0(0) / 0(0)<br>17(89.5) / 0(0) / 2(10.5)
Overall assessment<br>(n=18)	okdoc: 2/38 (5.3)<br>docapp: 1/57 (1.8)	$\chi^2_{(3)}$=4.0,<br>*P*=.26	2(100) / 0(0) / 0(0)<br>1(100) / 0(0) / 0(0)

	medicosearch: 2/234 (0.9)<br>google: 13/520 (2.5)		1(50) / 1(50) / 0(0)<br>12(92.3) / 0(0) / 1(7.7)
Professionalism<br>(n=15)	okdoc: 0/38 (0.0)<br>docapp: 1/57 (1.8)<br>medicosearch: 3/234 (1.3)<br>google: 11/520 (2.1)	$\chi^2_{(3)}$=1.3,<br>*P*=.71	0(0) / 0(0) / 0(0)<br>0(0) / 0(0) / 1(100)<br>2(66.7) / 1(33.3) / 0(0)<br>8(72.7) / 0(0) / 3(27.3)
Communication<br>(n=13)	okdoc: 0/38 (0.0)<br>docapp: 0/57 (0.0)<br>medicosearch: 1/234 (0.4)<br>google: 12/520 (2.3)	$\chi^2_{(3)}$=5.4,<br>*P*=.14	0(0) / 0(0) / 0(0)<br>0(0) / 0(0) / 0(0)<br>1(100) / 0(0) / 0(0)<br>4(33.3) / 1(8.3) / 7(58.3)
Availability by telephone<br>(n=3)	okdoc: 1/38 (2.6)<br>docapp: 2/57 (3.5)<br>medicosearch: 0/234 (0.0)<br>google: 0/520 (0.0)	**$\chi^2_{(3)}$=24.4,**<br>***P*<.001, V=.17**	1(100) / 0(0) / 0(0)<br>2(100) / 0(0) / 0(0)<br>0(0) / 0(0) / 0(0)<br>0(0) / 0(0) / 0(0)
Recommendation<br>(n=1)	okdoc: 0/38 (0.0)<br>docapp: 0/57 (0.0)<br>medicosearch: 0/234 (0.0)<br>google: 1/520 (0.2)	$\chi^2_{(3)}$=.63,<br>*P*=.90	0(0) / 0(0) / 0(0)<br>0(0) / 0(0) / 0(0)<br>0(0) / 0(0) / 0(0)<br>1(100) / 0(0) / 0(0)
Time spent with patient<br>(n=1)	okdoc: 0/38 (0.0)<br>docapp: 0/57 (0.0)<br>medicosearch: 1/234 (0.4)<br>google: 0/520 (0.0)	$\chi^2_{(3)}$=2.6,<br>*P*=.45	0(0) / 0(0) / 0(0)<br>0(0) / 0(0) / 0(0)<br>1(100) / 0(0) / 0(0)<br>0(0) / 0(0) / 0(0)
**Practice (N=237)**			
Atmosphere<br>(n=59)	okdoc: 1/38 (2.6)<br>docapp: 7/57 (12.3)	$\chi^2_{(3)}$=6.9,<br>*P*=.08	1(100) / 0(0) / 0(0)<br>7(100) / 0(0) / 0(0)

	medicosearch: 10/234 (4.3)<br>google: 41/520 (7.9)		8(80) / 2(20) / 0(0)<br>38(92.7) / 1(2.4) / 2(4.9)
Waiting time within practice<br>(n=58)	okdoc: 2/38 (5.3)<br>docapp: 7/57 (12.3)<br>medicosearch: 14/234 (6.0)<br>google: 35/520 (6.7)	$\chi^2_{(3)}$=3.1,<br>*P*=.38	2(100) / 0(0) / 0(0)<br>6(85.7) / 0(0) / 1(14.3)<br>9(64.3) / 1(7.1) / 4(28.6)<br>25(71.4) / 3(8.6) / 7(20)
Ability to get appointment<br>(n=39)	okdoc: 1/38 (2.6)<br>docapp: 3/57 (5.3)<br>medicosearch: 6/234 (2.6)<br>google: 29/520 (5.6)	$\chi^2_{(3)}$=3.7,<br>*P*=.29	1(100) / 0(0) / 0(0)<br>3(100) / 0(0) / 0(0)<br>6(100) / 0(0) / 0(0)<br>21(72.4) / 0(0) / 8(27.6)
Overall assessment<br>(n=22)	okdoc: 2/38 (5.3)<br>docapp: 2/57 (3.5)<br>medicosearch: 7/234 (3.0)<br>google: 11/520 (2.1)	$\chi^2_{(3)}$=1.8,<br>*P*=.60	2(100) / 0(0) / 0(0)<br>2(100) / 0(0) / 0(0)<br>6(85.7) / 1(14.3) / 0(0)<br>10(90.9) / 0(0) / 1(9.1)
Location<br>(n=15)	okdoc: 1/38 (2.6)<br>docapp: 2/57 (3.5)<br>medicosearch: 2/234 (0.9)<br>google: 10/520 (1.9)	$\chi^2_{(3)}$=2.4,<br>*P*=.50	1(100) / 0(0) / 0(0)<br>2(100) / 0(0) / 0(0)<br>2(100) / 0(0) / 0(0)<br>8(80) / 0(0) / 2(20)
Organisation<br>(n=13)	okdoc: 0/38 (0.0)<br>docapp: 3/57 (5.3)<br>medicosearch: 3/234 (1.3)<br>google: 7/520 (1.3)	$\chi^2_{(3)}$=6.1,<br>*P*=.11	0(0) / 0(0) / 0(0)<br>3(100) / 0(0) / 0(0)<br>3(100) / 0(0) / 0(0)<br>4(57.1) / 1(14.3) / 2(28.6)
Equipment<br>(n=9)	okdoc: 0/38 (0.0)<br>docapp: 0/57 (0.0)<br>medicosearch: 0/234 (0.0)	$\chi^2_{(3)}$=5.8,<br>*P*=.12	0(0) / 0(0) / 0(0)<br>0(0) / 0(0) / 0(0)<br>0(0) / 0(0) / 0(0)

	google: 9/520 (1.7)		8(88.9) / 0(0) / 1(11.1)
Online appointment<br>(n=5)	okdoc: 0/38 (0.0)<br>docapp: 0/57 (0.0)<br>medicosearch: 4/234 (1.7)<br>google: 1/520 (0.2)	$\chi^2_{(3)}$=7.0,<br>*P*=.07	0(0) / 0(0) / 0(0)<br>0(0) / 0(0) / 0(0)<br>4(100) / 0(0) / 0(0)<br>1(100) / 0(0) / 0(0)
Recommendation<br>(n=5)	okdoc: 0/38 (0.0)<br>docapp: 1/57 (1.8)<br>medicosearch: 1/234 (0.4)<br>google: 3/520 (0.6)	$\chi^2_{(3)}$=1.7,<br>*P*=.65	0(0) / 0(0) / 0(0)<br>1(100) / 0(0) / 0(0)<br>1(100) / 0(0) / 0(0)<br>3(100) / 0(0) / 0(0)
Parking space<br>(n=5)	okdoc: 0/38 (0.0)<br>docapp: 1/57 (1.8)<br>medicosearch: 0/234 (0.0)<br>google: 4/520 (0.8)	$\chi^2_{(3)}$=3.2,<br>*P*=.36	0(0) / 0(0) / 0(0)<br>1(100) / 0(0) / 0(0)<br>0(0) / 0(0) / 0(0)<br>4(100) / 0(0) / 0(0)
Consultation hours<br>(n=2)	okdoc: 0/38 (0.0)<br>docapp: 0/57 (0.0)<br>medicosearch: 2/234 (0.9)<br>google: 0/520 (0.0)	$\chi^2_{(3)}$=5.3,<br>*P*=.15	0(0) / 0(0) / 0(0)<br>0(0) / 0(0) / 0(0)<br>2(100) / 0(0) / 0(0)<br>0(0) / 0(0) / 0(0)
Waiting room entertainment<br>(n=2)	okdoc: 0/38 (0.0)<br>docapp: 0/57 (0.0)<br>medicosearch: 0/234 (0.0)<br>google: 2/520 (0.4)	$\chi^2_{(3)}$=1.3,<br>*P*=.74	0(0) / 0(0) / 0(0)<br>0(0) / 0(0) / 0(0)<br>0(0) / 0(0) / 0(0)<br>2(100) / 0(0) / 0(0)
Availability by telephone<br>(n=2)	okdoc: 0/38 (0.0)<br>docapp: 0/57 (0.0)<br>medicosearch: 0/234 (0.0)<br>google: 2/520 (0.4)	$\chi^2_{(3)}$=1.3,<br>*P*=.74	0(0) / 0(0) / 0(0)<br>0(0) / 0(0) / 0(0)<br>0(0) / 0(0) / 0(0)<br>1(50) / 0(0) / 1(50)

Barrier free access	okdoc: 0/38 (0.0)	$\chi^2_{(3)}$=.63,	0(0) / 0(0) / 0(0)
(n=1)	docapp: 0/57 (0.0)	*P*=.90	0(0) / 0(0) / 0(0)
	medicosearch: 0/234 (0.0)		0(0) / 0(0) / 0(0)
	google: 1/520 (0.1)		0(0) / 1(100) / 0(0)

3
