# Supplementary material for: The Content and Nature of Narrative Comments on Swiss Physician Rating Websites: Analysis of 849 Comments
Source: J Med Internet Res. 2019 Sep 30;21(9):e14336. doi: 10.2196/14336 (PMC6792026; doi:10.2196/14336)
Supplement: Multimedia Appendix 2 [file jmir_v21i9e14336_app2.pdf]

Multimedia Appendix 2. Categorisation of issues by regions

Issue	Region (%)	Chi-squared-test	Evaluation<br>% (+/=/-)
**Physician (N=2042)**			
Overall assessment (n=300)	Zurich: 223/668 (33.4)<br>Geneva: 77/181 (42.5)	**$\chi^2_{(1)}$=5.2,**<br>***P*=.03**	205(91.9) / 5(2.2) / 13(5.8)<br>73(94.8) / 2(2.6) / 2(2.6)
Competence<br>(n=300)	Zurich: 263/668 (39.4)<br>Geneva: 37/181 (20.4)	**$\chi^2_{(1)}$=22.3,**<br>***P*<.001**	251(95.4) / 4(1.5) / 8(3.0)<br>33(89.2) / 1(2.7) / 3(8.1)
Communication<br>(n=232)	Zurich: 172/668 (25.7)<br>Geneva: 60/181 (33.1)	**$\chi^2_{(1)}$=3.9,**<br>***P*=.049**	148(86.0) / 2(1.2) / 22(12.8)<br>49(81.7) / 0(0) / 11(18.3)
Recommendation<br>(n=225)	Zurich: 178/668 (26.6)<br>Geneva: 47/181 (26.0)	$\chi^2_{(1)}$=.03,<br>*P*=.92	159(89.3) / 0(0) / 19(10.7)<br>35(74.5) / 0(0) / 12(25.5)
Friendliness<br>(n=215)	Zurich: 175/668 (26.2)<br>Geneva: 40/181 (22.1)	$\chi^2_{(1)}$=1.3,<br>*P*=.29	154(88.0) / 4(2.3) / 17(9.7)<br>37(92.5) / 1(2.5) / 2(5.0)
Caring attitude<br>(n=192)	Zurich: 142/668 (21.3)<br>Geneva: 50/181 (27.6)	$\chi^2_{(1)}$=3.3,<br>*P*=.07	122(85.9) / 3(2.1) / 17(12.0)<br>46(92) / 0(0) / 4(8.0)
Satisfaction with treatment<br>(n=149)	Zurich: 122/668 (18.3)<br>Geneva: 27/181 (14.9)	$\chi^2_{(1)}$=1.1,<br>*P*<.32	98(80.3) / 4(3.3) / 20(16.4)<br>20(74.1) / 0(0) / 7(25.9)
Professionalism<br>(n=129)	Zurich: 90/668 (13.5)<br>Geneva: 39/181 (21.5)	**$\chi^2_{(1)}$=7.2,**<br>***P*=.01**	68(75.6) / 4(4.4) / 18(20)<br>31(79.5) / 0(0) / 8(20.5)
Time spent with patient<br>(n=107)	Zurich: 93/668 (13.9)<br>Geneva: 14/181 (7.7)	**$\chi^2_{(1)}$=5.0,**<br>***P*=.03**	82(88.2) / 2(2.2) / 9(9.7)<br>12(85.7) / 0(0) / 2(14.3)
Trust<br>(n=82)	Zurich: 58/668 (8.7)<br>Geneva: 24/181 (13.3)	$\chi^2_{(1)}$=3.4,<br>*P*=.09	55(94.8) / 0(0) / 3(5.2)<br>18(75.0) / 0(0) / 6(25.0)
Treatment cost/billing	Zurich: 30/668 (4.5)	$\chi^2_{(1)}$=2.1,	7(23.3) / 1(3.3) / 22(73.3)

(n=43)	Geneva: 13/181 (7.2)	*P*=.18	3(23.1) / 0(0) / 10(76.9)
Being taken seriously<br>(n=30)	Zurich: 30/668 (4.5)<br>Geneva: 0/181 (0.0)	**$\chi^2_{(1)}$=8.4,<br>*P*=.001**	25(83.3) / 0(0) / 5(16.7)<br>0(0) / 0(0) / 0(0)
Cooperation with medical specialists<br>(n=11)	Zurich: 9/668 (1.3)<br>Geneva: 2/181 (1.1)	$\chi^2_{(1)}$=.07,<br>*P*=1.0	9(100) / 0(0) / 0(0)<br>2(100) / 0(0) / 0(0)
Alternative medicine<br>(n=5)	Zurich: 3/668 (0.4)<br>Geneva: 2/181 (1.1)	$\chi^2_{(1)}$=1.1,<br>*P*=.29	3(100) / 0(0) / 0(0)<br>2(100) / 0(0) / 0(0)
Patient involvement<br>(n=5)	Zurich: 4/668 (0.6)<br>Geneva: 1/181 (0.6)	$\chi^2_{(1)}$=.005,<br>*P*=1.0	4(100) / 0(0) / 0(0)<br>1(100) / 0(0) / 0(0)
Telephone availability<br>(n=5)	Zurich: 3/668 (0.4)<br>Geneva: 2/181 (1.1)	$\chi^2_{(1)}$=1.1,<br>*P*=.30	2(66.7) / 0(0) / 1(33.3)<br>2(100) / 0(0) / 0(0)
Individualised service<br>(n=4)	Zurich: 3/668 (0.4)<br>Geneva: 1/181 (0.6)	$\chi^2_{(1)}$=.03,<br>*P*=1.0	3(100) / 0(0) / 0(0)<br>1(100) / 0(0) / 0(0)
House visits<br>(n=3)	Zurich: 1/668 (0.1)<br>Geneva: 2/181 (1.1)	$\chi^2_{(1)}$=3.7,<br>*P*=.12	1(100) / 0(0) / 0(0)<br>2(100) / 0(0) / 0(0)
Available outside normal hours<br>(n=2)	Zurich: 2/668 (0.3)<br>Geneva: 0/181 (0.0)	$\chi^2_{(1)}$=.54,<br>*P*=1.0	2(100) / 0(0) / 0(0)<br>0(0) / 0(0) / 0(0)
Privacy<br>(n=2)	Zurich: 2/668 (0.3)<br>Geneva: 0/181 (0.0)	$\chi^2_{(1)}$=.54,<br>*P*=1.0	2(100) / 0(0) / 0(0)<br>0(0) / 0(0) / 0(0)
Health insurance differentiation<br>(n=1)	Zurich: 0/668 (0.0)<br>Geneva: 1/181 (0.6)	$\chi^2_{(1)}$=3.7,<br>*P*=.21	0(0) / 0(0) / 0(0)<br>0(0) / 0(0) / 1(100)
**Staff (N=162)**			
Friendliness<br>(n=92)	Zurich: 78/668 (11.7)<br>Geneva: 14/181 (7.7)	$\chi^2_{(1)}$=2.3,<br>*P*=.14	66(84.6) / 6(7.7) / 6(7.7)<br>12(85.7) / 0(0) / 2(14.3)

Service/assistance<br>(n=19)	Zurich: 13/668 (1.9)<br>Geneva: 6/181 (3.3)	$\chi^2_{(1)}$=1.2,<br>*P*=.26	12(92.3) / 0(0) / 1(7.7)<br>5(83.3) / 0(0) / 1(16.7)
Overall assessment<br>(n=18)	Zurich: 12/668 (1.8)<br>Geneva: 6/181 (3.3)	$\chi^2_{(1)}$=1.6,<br>*P*=.24	11(91.7) / 0(0) / 1(8.3)<br>5(83.3) / 0(0) / 1(16.7)
Professionalism<br>(n=15)	Zurich: 14/668 (2.1)<br>Geneva: 1/181 (0.6)	$\chi^2_{(1)}$=2.0,<br>*P*=.21	10(71.4) / 1(7.1) / 3(21.4)<br>0(0) / 0(0) / 1(100)
Communication<br>(n=13)	Zurich: 10/668 (1.5)<br>Geneva: 3/181 (1.7)	$\chi^2_{(1)}$=.02,<br>*P*=.75	4(40) / 1(10) / 5(50)<br>1(33.3) / 0(0) / 2(66.7)
Availability by telephone<br>(n=3)	Zurich: 3/668 (0.4)<br>Geneva: 0/181 (0.0)	$\chi^2_{(1)}$=.82,<br>*P*=1.0	3(100) / 0(0) / 0(0)<br>0(0) / 0(0) / 0(0)
Recommendation<br>(n=1)	Zurich: 1/668 (0.1)<br>Geneva: 0/181 (0.0)	$\chi^2_{(1)}$=.27,<br>*P*=1.0	1(100) / 0(0) / 0(0)<br>0(0) / 0(0) / 0(0)
Time spent with patient<br>(n=1)	Zurich: 1/668 (0.1)<br>Geneva: 0/181 (0.0)	$\chi^2_{(1)}$=.27,<br>*P*=1.0	1(100) / 0(0) / 0(0)<br>0(0) / 0(0) / 0(0)
**Practice (N=237)**			
Atmosphere<br>(n=59)	Zurich: 47/668 (7.0)<br>Geneva: 12/181 (6.6)	$\chi^2_{(1)}$=.04,<br>*P*=1.0	43(91.5) / 3(6.5) / 1(2.1)<br>11(91.7) / 0(0) / 1(8.3)
Waiting time within practice<br>(n=58)	Zurich: 54/668 (8.1)<br>Geneva: 4/181 (2.2)	**$\chi^2_{(1)}$=7.7,**<br>***P*=.004**	38(70.4) / 4(7.4) / 12(22.2)<br>4(100) / 0(0) / 0(0)
Ability to get appointment<br>(n=39)	Zurich: 34/668 (5.1)<br>Geneva: 5/181 (2.8)	$\chi^2_{(1)}$=1.8,<br>*P*=.23	28(82.4) / 0(0) / 6(17.6)<br>3(60) / 0(0) / 2(40)
Overall assessment<br>(n=22)	Zurich: 20/668 (3.0)<br>Geneva: 2/181 (1.1)	$\chi^2_{(1)}$=2.0,<br>*P*=.19	18(90) / 1(5.0) / 1(5.0)<br>2(100) / 0(0) / 0(0)
Location<br>(n=15)	Zurich: 13/668 (1.9)<br>Geneva: 2/181 (1.1)	$\chi^2_{(1)}$=.58,<br>*P*=.75	11(84.6) / 0(0) / 2(15.4)<br>2(100) / 0(0) / 0(0)

Organisation<br>(n=13)	Zurich: 13/668 (1.9)<br>Geneva: 0/181 (0.0)	$\chi^2_{(1)}$=3.6,<br>*P*=.08	10(76.9) / 1(7.7) / 2(15.4)<br>0(0) / 0(0) / 0(0)
Equipment<br>(n=9)	Zurich: 3/668 (0.4)<br>Geneva: 6/181 (3.3)	**$\chi^2_{(1)}$=11.2,**<br>***P*=.004**	3(100) / 0(0) / 0(0)<br>5(83.3) / 0(0) / 1(16.7)
Online appointment<br>(n=5)	Zurich: 4/668 (0.6)<br>Geneva: 1/181 (0.6)	$\chi^2_{(1)}$=.005,<br>*P*=1.0	4(100) / 0(0) / 0(0)<br>1(100) / 0(0) / 0(0)
Recommendation<br>(n=5)	Zurich: 4/668 (0.6)<br>Geneva: 1/181 (0.6)	$\chi^2_{(1)}$=.005,<br>*P*=1.0	4(100) / 0(0) / 0(0)<br>1(100) / 0(0) / 0(0)
Parking space<br>(n=5)	Zurich: 2/668 (0.3)<br>Geneva: 3/181 (1.7)	$\chi^2_{(1)}$=4.5,<br>*P*=.07	2(100) / 0(0) / 0(0)<br>3(100) / 0(0) / 0(0)
Consultation hours<br>(n=2)	Zurich: 2/668 (0.3)<br>Geneva: 0/181 (0.0)	$\chi^2_{(1)}$=.54,<br>*P*=1.0	2(100) / 0(0) / 0(0)<br>0(0) / 0(0) / 0(0)
Waiting room entertainment<br>(n=2)	Zurich: 1/668 (0.1)<br>Geneva: 1/181 (0.6)	$\chi^2_{(1)}$=.98,<br>*P*=.38	1(100) / 0(0) / 0(0)<br>1(100) / 0(0) / 0(0)
Availability by telephone<br>(n=2)	Zurich: 1/668 (0.1)<br>Geneva: 1/181 (0.6)	$\chi^2_{(1)}$=.98,<br>*P*=.38	1(100) / 0(0) / 0(0)<br>0(0) / 0(0) / 1(100)
Barrier free access<br>(n=1)	Zurich: 0/668 (0.0)<br>Geneva: 1/181 (0.6)	$\chi^2_{(1)}$=3.7,<br>*P*=.21	0(0) / 0(0) / 0(0)<br>0(0) / 1(100) / 0(0)
