# Supplementary material for: The Content and Nature of Narrative Comments on Swiss Physician Rating Websites: Analysis of 849 Comments
Source: J Med Internet Res. 2019 Sep 30;21(9):e14336. doi: 10.2196/14336 (PMC6792026; doi:10.2196/14336)
Supplement: Multimedia Appendix 3 [file jmir_v21i9e14336_app3.pdf]

Multimedia Appendix 3. Categorisation of issues by speciality

Issue	Speciality	Chi-squared-test	Evaluation<br>% (+/=/-)
**Physician (N=2042)**			
Overall assessment (n=300)	GP: 112/306 (36.6)<br>Specialist: 188/543 (34.6)	$\chi^2_{(1)}$=.34,<br>*P*=.60	103(92.0) / 2(1.8) / 7(6.3)<br>175(93.1) / 5(2.7) / 8(4.3)
Competence (n=300)	GP: 109/306 (35.6)<br>Specialist: 191/543 (35.2)	$\chi^2_{(1)}$=.02,<br>*P*=.94	104(95.4) / 2(1.8) / 3(2.8)<br>180(94.2) / 3(1.6) / 8(4.2)
Communication (n=232)	GP: 94/306 (30.7)<br>Specialist: 138/543 (25.4)	$\chi^2_{(1)}$=2.8,<br>*P*=.11	88(93.6) / 1(1.1) / 5(5.3)<br>109(79.0)/ 1(0.7) / 28(20.3)
Recommendation (n=225)	GP: 63/306 (20.6)<br>Specialist: 162/543 (29.8)	**$\chi^2_{(1)}$=8.6,**<br>***P*=.004**	55(87.3) / 0(0) / 8(12.7)<br>139(85.8) / 0(0) / 23(14.2)
Friendliness (n=215)	GP: 74/306 (24.2)<br>Specialist: 141/543 (26.0)	$\chi^2_{(1)}$=.33,<br>*P*=.62	68(91.9) / 3(4.1) / 3(4.1)<br>123(87.2) / 2(1.4) / 16(11.3)
Caring attitude (n=192)	GP: 52/306 (17.0)<br>Specialist: 140/543 (25.8)	**$\chi^2_{(1)}$=8.6,**<br>***P*=.004**	50(96.2) / 1(1.9) / 1(1.9)<br>118(84.3)/ 2(1.4) / 20(14.3)
Satisfaction with treatment (n=149)	GP: 37/306 (12.1)<br>Specialist: 112/543 (20.6)	**$\chi^2_{(1)}$=9.9,**<br>***P*=.002**	32(86.5) / 2(5.4) / 3(8.1)<br>86(76.8) / 2(1.8) / 24(21.4)
Professionalism (n=129)	GP: 40/306 (13.1)<br>Specialist: 89/543 (16.4)	$\chi^2_{(1)}$=1.7,<br>*P*=.232	34(85.0) / 1(2.5) / 5(12.5)<br>65(73.0) / 3(3.4) / 21(23.6)
Time spent with patient (n=107)	GP: 42/306 (13.7)<br>Specialist: 65/543 (12.0)	$\chi^2_{(1)}$=.55,<br>*P*=.45	41(97.6) / 0(0) / 1(2.4)<br>53(81.5) / 2(3.1) / 10(15.4)
Trust (n=82)	GP: 30/306 (9.8)<br>Specialist: 52/543 (9.6)	$\chi^2_{(1)}$=.01,<br>*P*=.90	29(96.7) / 0(0) / 1(3.3)<br>44(84.6) / 0(0) / 8(15.4)
Treatment cost/billing (n=43)	GP: 6/306 (2.0)<br>Specialist: 37/543 (6.8)	**$\chi^2_{(1)}$=9.6,**	0(0) / 1(16.7) / 5(83.3)<br>10(27.0) / 0(0) / 27(73)

		***P*=.002**	
Being taken seriously<br>(n=30)	GP: 13/306 (4.2)<br>Specialist: 17/543 (3.1)	$\chi^2_{(1)}$=.72,<br>*P*=.44	10(76.9) / 0(0) / 3(23.1)<br>15(88.2) / 0(0) / 2(11.8)
Cooperation with medical specialists<br>(n=11)	GP: 9/306 (2.9)<br>Specialist: 2/543 (0.4)	**$\chi^2_{(1)}$=10.1,**<br>***P*=.002**	9(100) / 0(0) / 0(0)<br>2(100) / 0(0) / 0(0)
Alternative medicine<br>(n=5)	GP: 4/306 (1.3)<br>Specialist: 1/543 (0.2)	$\chi^2_{(1)}$=4.2,<br>*P*=.06	4(100) / 0(0) / 0(0)<br>1(100) / 0(0)/ 0(0)
Patient involvement<br>(n=5)	GP: 4/306 (1.3)<br>Specialist: 1/543 (0.2)	$\chi^2_{(1)}$=4.2,<br>*P*=.06	4(100) / 0(0) / 0(0)<br>1(100) / 0(0)/ 0(0)
Telephone availability<br>(n=5)	GP: 1/306 (0.3)<br>Specialist: 4/543 (0.7)	$\chi^2_{(1)}$=.56,<br>*P*=.66	1(100) / 0(0)/ 0(0)<br>3(75) / 0(0) / 1(25)
Individualised service<br>(n=4)	GP: 1/306 (0.3)<br>Specialist: 3/543 (0.6)	$\chi^2_{(1)}$=.21,<br>*P*=1.0	1(100) / 0(0)/ 0(0)<br>3(100) / 0(0)/ 0(0)
House visits<br>(n=3)	GP: 3/306 (1.0)<br>Specialist: 0/543 (0.0)	**$\chi^2_{(1)}$=5.3,**<br>***P*=.047**	3(100) / 0(0)/ 0(0)<br>0(0) / 0(0) / 0(0)
Available outside normal hours<br>(n=2)	GP: 0/306 (0.0)<br>Specialist: 2/543 (0.4)	$\chi^2_{(1)}$=1.1,<br>*P*=.54	0(0) / 0(0) / 0(0)<br>2(100) / 0(0) / 0(0)
Privacy<br>(n=2)	GP: 0/306 (0.0)<br>Specialist: 2/543 (0.4)	$\chi^2_{(1)}$=1.1,<br>*P*=.54	0(0) / 0(0) / 0(0)<br>2(100) / 0(0) / 0(0)
Health insurance differentiation<br>(n=1)	GP: 0/306 (0.0)<br>Specialist: 1/543 (0.2)	$\chi^2_{(1)}$=.56,<br>*P*=1.0	0(0) / 0(0) / 0(0)<br>0(0) / 0(0) / 1(100)
**Staff (N=162)**			
Friendliness<br>(n=92)	GP: 18/306 (5.9)<br>Specialist: 74/543 (13.6)	**$\chi^2_{(1)}$=12.2,**<br>***P*<.001**	16(88.9) / 1(5.6) / 1(5.6)<br>62(83.8) / 5(6.8) / 7(9.5)

Service/assistance<br>(n=19)	GP: 3/306 (1.0)<br>Specialist: 16/543 (2.9)	$\chi^2_{(1)}$=3.5,<br>*P*=.09	2(66.7) / 0(0) / 1(33.3)<br>15(93.8) / 0(0) / 1(6.3)
Overall assessment<br>(n=18)	GP: 6/306 (2.0)<br>Specialist: 12/543 (2.2)	$\chi^2_{(1)}$=.06,<br>*P*=1.0	6(100) / 0(0) / 0(0)<br>10(83.3) / 1(8.3) / 1(8.3)
Professionalism<br>(n=15)	GP: 4/306 (1.3)<br>Specialist: 11/543 (2.0)	$\chi^2_{(1)}$=.58,<br>*P*=.59	4(100) / 0(0) / 0(0)<br>6(54.5) / 1(9.1) / 4(36.4)
Communication<br>(n=13)	GP: 3/306 (1.0)<br>Specialist: 10/543 (1.8)	$\chi^2_{(1)}$=.96,<br>*P*=.40	1(33.3) / 0(0) / 2(66.7)<br>4(40) / 1(10) / 5(50)
Availability by telephone<br>(n=3)	GP: 2/306 (0.7)<br>Specialist: 1/543 (0.2)	$\chi^2_{(1)}$=1.2,<br>*P*=.30	2(100) / 0(0) / 0(0)<br>1(100) / 0(0) / 0(0)
Recommendation<br>(n=1)	GP: 1/306 (0.3)<br>Specialist: 0/543 (0.0)	$\chi^2_{(1)}$=1.8,<br>*P*=.36	1(100) / 0(0) / 0(0)<br>0(0) / 0(0) / 0(0)
Time spent with patient<br>(n=1)	GP: 0/306 (0.0)<br>Specialist: 1/543 (0.2)	$\chi^2_{(1)}$=.56,<br>*P*=1.0	0(0) / 0(0) / 0(0)<br>1(100) / 0(0) / 0(0)
**Practice (N=237)**			
Atmosphere<br>(n=59)	GP: 12/306 (3.9)<br>Specialist: 47/543 (8.7)	**$\chi^2_{(1)}$=6.8,**<br>***P*=.01**	12(100) / 0(0) / 0(0)<br>42(89.4) / 3(6.4) / 2(4.3)
Waiting time within practice<br>(n=58)	GP: 18/306 (5.9)<br>Specialist: 40/543 (7.4)	$\chi^2_{(1)}$=.68,<br>*P*=.48	15(83.3) / 0(0) / 3(16.7)<br>27(67.5) / 4(10) / 9(22.5)
Ability to get appointment<br>(n=39)	GP: 15/306 (4.9)<br>Specialist: 24/543 (4.4)	$\chi^2_{(1)}$=.10,<br>*P*=.74	13(86.7) / 0(0) / 2(13.3)<br>18(75) / 0(0) / 6(25)
Overall assessment<br>(n=22)	GP: 9/306 (2.9)<br>Specialist: 13/543 (2.4)	$\chi^2_{(1)}$=.23,<br>*P*=.66	8(88.9) / 0(0) / 1(11.1)<br>12(92.3) / 1(7.7) / 0(0)
Location<br>(n=15)	GP: 3/306 (1.0)<br>Specialist: 12/543 (2.2)	$\chi^2_{(1)}$=1.7,<br>*P*=.28	3(100) / 0(0) / 0(0)<br>10(83.3) / 0(0) / 2(16.7)

Organisation<br><br>(n=13)	GP: 3/306 (1.0)<br><br>Specialist: 10/543 (1.8)	$\chi^2_{(1)}$=.96,<br><br>*P*=.40	2(66.7) / 0(0) / 1(33.3)<br><br>8(80) / 1(10) / 1(10)
Equipment<br><br>(n=9)	GP: 0/306 (0.0)<br><br>Specialist: 9/543 (1.7)	$\chi^2_{(1)}$=5.1,<br><br>*P*=.03	0(0) / 0(0) / 0(0)<br><br>8(88.9) / 0(0) / 1(11.1)
Online appointment<br><br>(n=5)	GP: 3/306 (1.0)<br><br>Specialist: 2/543 (0.4)	$\chi^2_{(1)}$=1.3,<br><br>*P*=.36	3(100) / 0(0) / 0(0)<br><br>2(100) / 0(0) / 0(0)
Recommendation<br><br>(n=5)	GP: 3/306 (1.0)<br><br>Specialist: 2/543 (0.4)	$\chi^2_{(1)}$=1.3,<br><br>*P*=.36	3(100) / 0(0) / 0(0)<br><br>2(100) / 0(0) / 0(0)
Parking space<br><br>(n=5)	GP: 2/306 (0.7)<br><br>Specialist: 3/543 (0.6)	$\chi^2_{(1)}$=.03,<br><br>*P*=1.0	2(100) / 0(0) / 0(0)<br><br>3(100) / 0(0) / 0(0)
Consultation hours<br><br>(n=2)	GP: 1/306 (0.3)<br><br>Specialist: 1/543 (0.2)	$\chi^2_{(1)}$=.17,<br><br>*P*=1.0	1(100) / 0(0) / 0(0)<br><br>1(100) / 0(0) / 0(0)
Waiting room entertainment<br><br>(n=2)	GP: 0/306 (0.0)<br><br>Specialist: 2/543 (0.4)	$\chi^2_{(1)}$=1.1,<br><br>*P*=.54	0(0) / 0(0) / 0(0)<br><br>2(100) / 0(0) / 0(0)
Availability by telephone<br><br>(n=2)	GP: 0/306 (0.0)<br><br>Specialist: 2/543 (0.4)	$\chi^2_{(1)}$=1.1,<br><br>*P*=.54	0(0) / 0(0) / 0(0)<br><br>1(50) / 0(0) / 1(50)
Barrier free access<br><br>(n=1)	GP: 0/306 (0.0)<br><br>Specialist: 1/543 (0.2)	$\chi^2_{(1)}$=.56,<br><br>*P*=1.0	0(0) / 0(0) / 0(0)<br><br>0(0) / 1(100) / 0(0)
