# Supplementary material for: The Content and Nature of Narrative Comments on Swiss Physician Rating Websites: Analysis of 849 Comments
Source: J Med Internet Res. 2019 Sep 30;21(9):e14336. doi: 10.2196/14336 (PMC6792026; doi:10.2196/14336)
Supplement: Multimedia Appendix 4 [file jmir_v21i9e14336_app4.pdf]

Multimedia Appendix 4. Categorisation of issues by gender

Issue	Gender (%)	Chi-squared-test	Evaluation % (+/=/-)
**Physician (N=2042)**			
Overall assessment<br>(n=300)	Male: 168/477 (35.2)<br>Female: 132/372 (35.5)	$\chi^2_{(1)}$=.01,<br>*P*=.94	154(91.7) / 3(1.8) / 11(6.5)<br>124(93.9) / 4(3.0) / 4(3.0)
Competence<br>(n=300)	Male: 161/477(33.8)<br>Female: 139/372(37.4)	$\chi^2_{(1)}$=1.2,<br>*P*=.28	154(95.7) /3(1.9) / 4(2.5)<br>130(93.5) / 2(1.4) / 7(5.0)
Communication<br>(n=232)	Male: 116/477 (24.3)<br>Female: 116/372 (31.2)	$\chi^2_{(1)}$=5.0,<br>*P*=.03	99(85.3) / 1(0.9) / 16(13.8)<br>98(84.5) / 1(0.9) / 17(14.7)
Recommendation<br>(n=225)	Male: 125/477 (26.2)<br>Female: 100/372 (26.9)	$\chi^2_{(1)}$=.05,<br>*P*=.90	110(88.0) / 0(0) / 15(12.0)<br>84(84.0) / 0(0) / 16(16.0)
Friendliness<br>(n=215)	Male: 115/477 (24.1)<br>Female: 100/372 (26.9)	$\chi^2_{(1)}$=.85,<br>*P*=.40	99(86.1) / 4(3.5) / 12(10.4)<br>92(92.0) / 1 (1.0) / 7(7.0)
Caring attitude<br>(n=192)	Male: 106/477 (22.2)<br>Female: 86/372 (23.1)	$\chi^2_{(1)}$=.10,<br>*P*=.80	95(89.6) / 2(1.9) / 9(8.5)<br>73(84.9) / 1(1.2) / 12(14.0)
Satisfaction with treatment<br>(n=149)	Male: 102/477 (21.4)<br>Female: 47/372 (12.6)	**$\chi^2_{(1)}$=11.1,**<br>***P*=.001**	82(80.4) / 4(3.9) / 16(15.7)<br>36(76.6) / 0(0) / 11(23.4)
Professionalism<br>(n=129)	Male: 67/477 (14.0)<br>Female: 62/372 (16.7)	$\chi^2_{(1)}$=1.1,<br>*P*=.30	51(76.1) / 2(3.0) / 14(20.9)<br>48(77.4) / 2(3.2) / 12(19.4)
Time spent with patient<br>(n=107)	Male: 64/477 (13.4)<br>Female: 43/372 (11.6)	**$\chi^2_{(1)}$=.65,**<br>***P*=.46**	57(89.1) / 2(3.1) / 5(7.8)<br>37(86.0) / 0(0) / 6(14.0)
Trust<br>(n=82)	Male: 43/477 (9.0)<br>Female: 39/372 (10.5)	$\chi^2_{(1)}$=.52,<br>*P*=.50	38(88.4) / 0(0) / 5(11.6)<br>35(89.7) / 0(0) / 4(10.3)

Treatment cost/billing<br>(n=43)	Male: 27/477 (5.7)<br>Female: 16/372 (4.3)	$\chi^2_{(1)}$=.80,<br>*P*=.43	9(33.3) / 1(3.7) / 17(63)<br>1(6.3) / 0(0) / 15(93.8)
Being taken seriously<br>(n=30)	Male: 8/477 (1.7)<br>Female: 22/372 (5.9)	**$\chi^2_{(1)}$=11.0,**<br>***P*=.001**	5(62.5) / 0(0) / 3(37.5)<br>20(90.9) / 0(0) / 2(9.1)
Cooperation with medical specialists<br>(n=11)	Male: 6/477 (1.3)<br>Female: 5/372 (1.3)	$\chi^2_{(1)}$=.01,<br>*P*=1.0	6(100) / 0(0) / 0(0)<br>5(100) / 0(0) / 0(0)
Alternative medicine<br>(n=5)	Male: 3/477 (0.6)<br>Female: 2/372 (0.5)	$\chi^2_{(1)}$=.03,<br>*P*=.62	3(100) / 0(0) / 0(0)<br>2(100) / 0(0) / 0(0)
Patient involvement<br>(n=5)	Male: 3/477 (0.6)<br>Female: 2/372 (0.5)	$\chi^2_{(1)}$=.03,<br>*P*=1.0	3(100) / 0(0) / 0(0)<br>2(100) / 0(0) / 0(0)
Telephone availability<br>(n=5)	Male: 5/477 (1.0)<br>Female: 0/372 (0.0)	$\chi^2_{(1)}$=3.9,<br>*P*=.07	4(80) / 0(0) / 1(20)<br>0(0) / 0(0) / 0(0)
Individualised service<br>(n=4)	Male: 2/477 (0.4)<br>Female: 2/372 (0.5)	$\chi^2_{(1)}$=.06,<br>*P*=1.0	2(100) / 0(0) / 0(0)<br>2(100) / 0(0) / 0(0)
House visits<br>(n=3)	Male: 3/477 (0.6)<br>Female: 0/372 (0.0)	$\chi^2_{(1)}$=2.3,<br>*P*=.30	3(100) / 0(0) / 0(0)<br>0(0) / 0(0) / 0(0)
Available outside normal hours<br>(n=2)	Male: 0/477 (0.0)<br>Female: 2/372 (0.5)	$\chi^2_{(1)}$=2.6,<br>*P*=.20	0(0) / 0(0) / 0(0)<br>2(100) / 0(0) / 0(0)
Privacy<br>(n=2)	Male: 0/477 (0.0)<br>Female: 2/372 (0.5)	$\chi^2_{(1)}$=2.6,<br>*P*=.20	0(0) / 0(0) / 0(0)<br>2(100) / 0(0) / 0(0)
Health insurance differentiation<br>(n=1)	Male: 1/477 (0.2)<br>Female: 0/372 (0.0)	$\chi^2_{(1)}$=.80,<br>*P*=1.0	0(0) / 0(0) / 1(100)<br>0(0) / 0(0) / 0(0)

**Staff (N=162)**			
Friendliness<br>(n=92)	Male: 55/477 (11.5)<br>Female: 37/372 (9.9)	$\chi^2_{(1)}$=.54,<br>P=.50	47(85.5) / 4(7.3) / 4(7.3)<br>31(83.8) / 2(5.4) / 4(10.8)
Service/assistance<br>(n=19)	Male: 17/477 (3.6)<br>Female: 2/372 (0.5)	**$\chi^2_{(1)}$=8.8,**<br>**P=.004**	15(88.2) / 0(0) / 2(11.8)<br>2(100) / 0(0) / (0)
Overall assessment<br>(n=18)	Male: 12/477 (2.5)<br>Female: 6/372 (1.6)	$\chi^2_{(1)}$=.82,<br>P=.50	10(83.3) / 1(8.3) / 1(8.3)<br>6(100) / 0(0) / (0)
Professionalism<br>(n=15)	Male: 7/477 (1.5)<br>Female: 8/372 (2.2)	$\chi^2_{(1)}$=.60,<br>P=.60	5(71.4) / 1(14.3) / 1(14.3)<br>5(62.5) / 0(0) / 3(37.5)
Communication<br>(n=13)	Male: 7/477 (1.5)<br>Female: 6/372 (1.6)	$\chi^2_{(1)}$=.03,<br>P=1.0	3(42.9) / 0(0) / 4(57.1)<br>2(33.3) / 1(16.7) / 3(50)
Availability by telephone<br>(n=3)	Male: 2/477 (0.4)<br>Female: 1/372 (0.3)	$\chi^2_{(1)}$=.13,<br>P=1.0	2(100) / 0(0) / (0)<br>1(100) / 0(0) / (0)
Recommendation<br>(n=1)	Male: 0/477 (0.0)<br>Female: 1/372 (0.3)	$\chi^2_{(1)}$=1.3,<br>P=.44	0(0) / 0(0) / (0)<br>1(100) / 0(0) / (0)
Time spent with patient<br>(n=1)	Male: 1/477 (0.2)<br>Female: 0/372 (0.0)	$\chi^2_{(1)}$=.80,<br>P=1.0	1(100) / 0(0) / (0)<br>0(0) / 0(0) / (0)
**Practice (N=237)**			
Atmosphere<br>(n=59)	Male: 26/477 (5.5)<br>Female: 33/372 (8.9)	$\chi^2_{(1)}$=3.8,<br>P=.06	24(92.3) / 2(7.7) / 0(0)<br>30(90.9) / 1(3.0) / 2(6.1)
Waiting time within practice<br>(n=58)	Male: 32/477 (6.7)<br>Female: 26/372 (7.0)	$\chi^2_{(1)}$=.03,<br>P=.90	24(75) / 1(3.1) / 7(21.9)<br>18(69.2) / 3(11.5) / 5(19.2)
Ability to get appointment<br>(n=39)	Male: 22/477 (4.6)<br>Female: 17/372 (4.6)	$\chi^2_{(1)}$=.001,<br>P=1.0	18(81.8) / 0(0) / 4(18.2)<br>13(76.5) / 0(0) / 4(23.5)

Overall assessment<br>(n=22)	Male: 12/477 (2.5)<br>Female: 10/372 (2.7)	$\chi^2_{(1)}$=.03,<br>P=1.0	10(83.3) / 1(8.3) / 1(8.3)<br>10(100) / 0(0) / (0)
Location<br>(n=15)	Male: 7/477 (1.5)<br>Female: 8/372 (2.2)	$\chi^2_{(1)}$=.60,<br>P=.60	6(85.7) / 0(0) / 1(14.3)<br>7(87.5) / 0(0) / 1(12.5)
Organisation<br>(n=13)	Male: 8/477 (1.7)<br>Female: 5/372 (1.3)	$\chi^2_{(1)}$=.20,<br>P=.80	5(62.5) / 1(12.5) / 2(25)<br>5(100) / 0(0) / (0)
Equipment<br>(n=9)	Male: 9/477 (1.9)<br>Female: 0/372 (0.0)	**$\chi^2_{(1)}$=7.1,**<br>**P=.006**	8(88.9) / 0(0) / 1(11.1)<br>0(0) / 0(0) / (0)
Online appointment<br>(n=5)	Male: 4/477 (0.8)<br>Female: 1/372 (0.3)	$\chi^2_{(1)}$=1.2,<br>P=.40	4(100) / 0(0) / (0)<br>1(100) / 0(0) / (0)
Recommendation<br>(n=5)	Male: 4/477 (0.8)<br>Female: 1/372 (0.3)	$\chi^2_{(1)}$=1.2,<br>P=.40	4(100) / 0(0) / (0)<br>1(100) / 0(0) / (0)
Parking space<br>(n=5)	Male: 3/477 (0.6)<br>Female: 2/372 (0.5)	$\chi^2_{(1)}$=.03,<br>P=1.0	3(100) / 0(0) / (0)<br>2(100) / 0(0) / (0)
Consultation hours<br>(n=2)	Male: 1/477 (0.2)<br>Female: 1/372 (0.3)	$\chi^2_{(1)}$=.03,<br>P=1.0	1(100) / 0(0) / (0)<br>1(100) / 0(0) / (0)
Waiting room entertainment<br>(n=2)	Male: 1/477 (0.2)<br>Female: 1/372 (0.3)	$\chi^2_{(1)}$=.03,<br>P=1.0	1(100) / 0(0) / (0)<br>1(100) / 0(0) / (0)
Availability by telephone<br>(n=2)	Male: 0/477 (0.0)<br>Female: 2/372 (0.5)	$\chi^2_{(1)}$=2.6,<br>P=.20	0(0) / 0(0) / (0)<br>1(50) / 0(0) / 1(50)
Barrier free access<br>(n=1)	Male: 0/477 (0.0)<br>Female: 1/372 (0.3)	$\chi^2_{(1)}$=1.3,<br>P=.44	0(0) / 0(0) / (0)<br>0(0) / 1(100) / 0(0)
